# 4-(Dimethyl­amino)­pyridinium *trans*-diaqua­bis­[oxalato(2−)-κ^2^ *O* ^1^,*O* ^2^]chromate(III)

**DOI:** 10.1107/S1600536810040353

**Published:** 2010-10-13

**Authors:** Justin Nenwa, Michel M. Belombe, Jean Ngoune, Boniface P. T. Fokwa

**Affiliations:** a Department of Inorganic Chemistry, University of Yaounde I, POB 812 Yaounde, Cameroon; b Department of Chemistry, University of Dschang, POB 67, Dschang, Cameroon; c Institut für Anorganische Chemie, RWTH Aachen, D-52056 Aachen, Germany

## Abstract

In the title salt, (C_7_H_11_N_2_)[Cr(C_2_O_4_)_2_(H_2_O)_2_], the asymmetric unit contains one half-cation and one half-anion. The Cr atom, the C and N atoms involved in C— N(exocyclic) bonding and the N and H atoms of N—H groups lie on twofold rotation axis. The Cr^III^ atom of the complex anion is six-coordinated in a distorted (4 + 2) octa­hedral geometry with four equatorial O atoms of two nearly coplanar oxalate and two quasi-axial aqua O atoms. In the crystal, the protonated N atoms of the pyridine rings are hydrogen bonded to the carbonyl O atoms of the anions, forming chains along [010]. These chains are connected by lateral O—H⋯O hydrogen bonds, stabilizing the structure.

## Related literature

For general background to the coordination chemistry of oxalate, see: Martin *et al.* (2007 ▶). For related structures, see: Bélombé *et al.* (2009 ▶); Ghouili *et al.* (2010 ▶). 
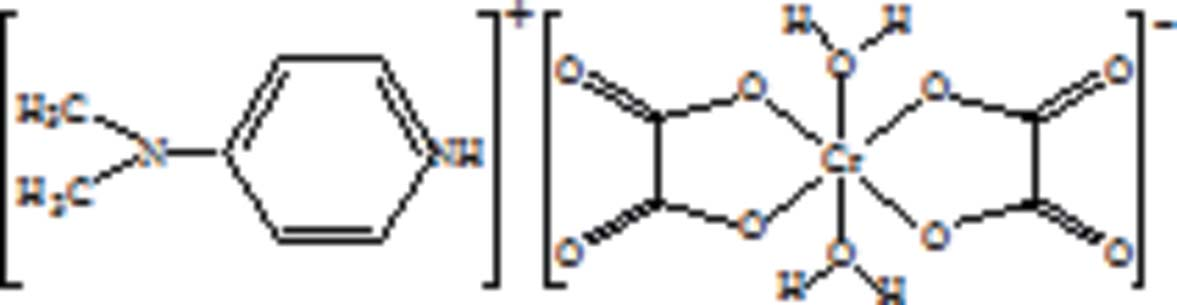


## Experimental

### 

#### Crystal data

(C_7_H_11_N_2_)[Cr(C_2_O_4_)_2_(H_2_O)_2_]
*M* *_r_* = 387.25
Monoclinic, 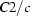

*a* = 11.524 (4) Å
*b* = 20.372 (8) Å
*c* = 7.355 (2) Å
β = 120.626 (6)°
*V* = 1485.9 (9) Å^3^
*Z* = 4
Mo *K*α radiation
μ = 0.83 mm^−1^
*T* = 293 K
0.20 × 0.20 × 0.10 mm

#### Data collection

Bruker APEX CCD area-detector diffractometer
Absorption correction: multi-scan (*SADABS*; Bruker, 2004 ▶) *T*_min_ = 0.895, *T*_max_ = 0.910
10203 measured reflections
1857 independent reflections
1739 reflections with *I* > 2σ(*I*)
*R*_int_ = 0.046

#### Refinement

*R*[*F* ^2^ > 2σ(*F* ^2^)] = 0.033
*wR*(*F* ^2^) = 0.089
*S* = 1.16
1857 reflections
127 parameters
2 restraints
H atoms treated by a mixture of independent and constrained refinement
Δρ_max_ = 0.37 e Å^−3^
Δρ_min_ = −0.38 e Å^−3^

### 

Data collection: *SMART* (Bruker, 2004 ▶); cell refinement: *SAINT* (Bruker, 2004 ▶); data reduction: *SAINT*; program(s) used to solve structure: *SHELXS97* (Sheldrick, 2008 ▶); program(s) used to refine structure: *SHELXL97* (Sheldrick, 2008 ▶); molecular graphics: *DIAMOND* (Brandenburg, 2010 ▶); software used to prepare material for publication: *WinGX* (Farrugia, 1999 ▶).

## Supplementary Material

Crystal structure: contains datablocks I, global. DOI: 10.1107/S1600536810040353/bx2312sup1.cif

Structure factors: contains datablocks I. DOI: 10.1107/S1600536810040353/bx2312Isup2.hkl

Additional supplementary materials:  crystallographic information; 3D view; checkCIF report

## Figures and Tables

**Table 1:** Hydrogen-bond geometry (Å, °)

*D*—H⋯*A*	*D*—H	H⋯*A*	*D*⋯*A*	*D*—H⋯*A*
O1—H1*A*⋯O4^i^	0.82 (1)	1.91 (1)	2.719 (2)	171 (2)
O1—H1*B*⋯O5^ii^	0.80 (1)	1.91 (1)	2.680 (2)	160 (2)
N12—H12⋯O4^iii^	0.86	2.19	2.906 (3)	141
N12—H12⋯O4^iv^	0.86	2.19	2.906 (3)	141

Symmetry codes: (i) 
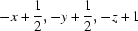
; (ii) 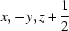; (iii) 
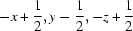
; (iv) 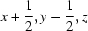.

**Fig. 1.**
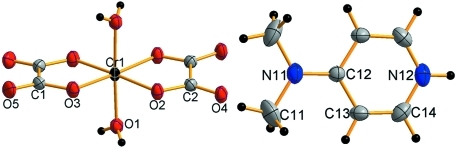
A view of the title compound with the atom-numbering scheme. Displacement ellipsoids are drawn at the 50% probability level and H atoms are shown as small spheres of arbitrary radii. Unlabelled atoms are related two labeled atoms by the symmetry code for cation and anion are: 1-x,y,1/2-z; -x,y,1/2-z

**Fig. 2.**
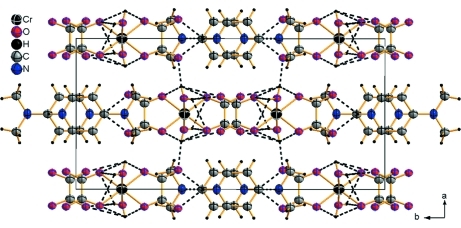
Packing diagram of the title compound. Dotted lines show hydrogen bonding.

## supplementary crystallographic information

### Comment

The coordination chemistry of oxalate (C_2_O_4_^2-^) continues receiving considerable attention, due to the ability of this ion to act as a remarkably flexible ligand system in complexations with a wide range of metal ions [Martin *et al.*,2007]. Recently, we published the structure of an organic-inorganic hybrid salt involving the quinolinium cation, [C_9_H_8_N]^+^and complex anion, [Cr(H_2_O)_2_(C_2_O_4_)_2_]^- ^(Bélombé *et al.*, 2009). We report here the crystal structure of a homologous salt, with 4-Dimethylaminopyridinium as the organic cation. The Fig. 1 shows the 4-dimethylaminopyridinium cation, [C_7_H_11_N_2_]^+^, and the complex anion, [Cr(H_2_O)_2_(C_2_O_4_)_2_]^-^.The asymmetric unit is formed by one-half cation and one-half anion. The geometrical parameters of the [C_7_H_11_N_2_]^+^ cation are in agreement with those found in salts with the same cationic entity (Ghouili *et al.*, 2010). The Cr^III^ ion of the complex anion adopts a distorted (4 + 2) octahedral coordination involving four equatorial O atoms (O2, O2^i^, O3, O3^i^) of two nearly coplanar oxalate and two quasi axial O atoms (O1, O1^i^) of water ligands (Fig. 1). The equatorial Cr–O distances are 1.9706 (13) Å (Cr–O(2), Cr–O(2^i^)) and 1.9468 (13) Å (Cr–O(3), Cr–O(3^i^)) respectively, and are significantly shorter than the axial Cr–O distance of 2.0055 (14)Å (Cr–O(1), Cr–O(1^i^)). The bond distances in the complex anion are comparable with those reported for the quinolinium compound (Bélombé *et al.*, 2009).In the crystal structure, intermolecular N–H···O(carbonyl) hydrogen bonds connect the ionic entities, generating layers parallel to [010]. These layers are further connected by lateral O–H···O hydrogen bonds,stabilizing the structure (Table 1, Fig. 2)

### Experimental

A mixture of 4-dimethylaminopyridine (1 mmol, 122.2 mg) and oxalic acid (2.2 mmol, 277.2 mg) was dissolved in 30 ml of water. The filtered solution was stirred at 328 K and an aqueous solution (20 ml) of CrCl_3_.6H_2_O (1 mmol, 266.5 mg) was added in successive small portions and stirred for 2 h continuously. The final red-violet solution obtained was left at room temperature and brown plate-like crystals suitable for X-ray diffraction were obtained after a few days.

### Refinement

The H atoms were positioned geometrically, with C—H, N—H distances of 0.96 and 0.86 Å respectively, and constrained to ride on their parent atoms, with *U*_iso_(H) = 1.5Ueq(C) and 1.2Ueq(N). The water H atoms were first located in a difference Fourier map and refined with distance restraints of d(O–H1) = 0.81 (1) with all *U*_iso_(H) refined freely.

### Crystal data

**Table:**

(C_7_H_11_N_2_)[Cr(C_2_O_4_)_2_(H_2_O)_2_]	*F*(000) = 796
*M**_r_* = 387.25	*D*_x_ = 1.731 Mg m^−^^3^
Monoclinic, *C*2/*c*	Mo *K*α radiation, λ = 0.71073 Å
Hall symbol: -C 2yc	Cell parameters from 1857 reflections
*a* = 11.524 (4) Å	θ = 2.0–28.3°
*b* = 20.372 (8) Å	µ = 0.83 mm^−^^1^
*c* = 7.355 (2) Å	*T* = 293 K
β = 120.626 (6)°	Prism, dark-violet
*V* = 1485.9 (9) Å^3^	0.20 × 0.20 × 0.10 mm
*Z* = 4	

### Data collection

**Table:**

Bruker APEX CCD area-detector diffractometer	1857 independent reflections
Radiation source: fine-focus sealed tube	1739 reflections with *I* > 2σ(*I*)
graphite	*R*_int_ = 0.046
integration method scans	θ_max_ = 28.3°, θ_min_ = 2.0°
Absorption correction: multi-scan (*SADABS*; Bruker, 2004)	*h* = −15→15
*T*_min_ = 0.895, *T*_max_ = 0.910	*k* = −27→27
10203 measured reflections	*l* = −9→9

### Refinement

**Table:**

Refinement on *F*^2^	Secondary atom site location: difference Fourier map
Least-squares matrix: full	Hydrogen site location: inferred from neighbouring sites
*R*[*F*^2^ > 2σ(*F*^2^)] = 0.033	H atoms treated by a mixture of independent and constrained refinement
*wR*(*F*^2^) = 0.089	*w* = 1/[σ^2^(*F*_o_^2^) + (0.0395*P*)^2^ + 1.1284*P*] where *P* = (*F*_o_^2^ + 2*F*_c_^2^)/3
*S* = 1.16	(Δ/σ)_max_ < 0.001
1857 reflections	Δρ_max_ = 0.37 e Å^−^^3^
127 parameters	Δρ_min_ = −0.38 e Å^−^^3^
2 restraints	Extinction correction: *SHELXL97* (Sheldrick, 2008), Fc^*^=kFc[1+0.001xFc^2^λ^3^/sin(2θ)]^-1/4^
Primary atom site location: structure-invariant direct methods	Extinction coefficient: 0.0065 (6)

### Special details

**Table:**

Geometry. All e.s.d.'s (except the e.s.d. in the dihedral angle between two l.s. planes) are estimated using the full covariance matrix. The cell e.s.d.'s are taken into account individually in the estimation of e.s.d.'s in distances, angles and torsion angles; correlations between e.s.d.'s in cell parameters are only used when they are defined by crystal symmetry. An approximate (isotropic) treatment of cell e.s.d.'s is used for estimating e.s.d.'s involving l.s. planes.
Refinement. Refinement of *F*^2^ against ALL reflections. The weighted *R*-factor *wR* and goodness of fit *S* are based on *F*^2^, conventional *R*-factors *R* are based on *F*, with *F* set to zero for negative *F*^2^. The threshold expression of *F*^2^ > σ(*F*^2^) is used only for calculating *R*-factors(gt) *etc*. and is not relevant to the choice of reflections for refinement. *R*-factors based on *F*^2^ are statistically about twice as large as those based on *F*, and *R*- factors based on ALL data will be even larger.

### Fractional atomic coordinates and isotropic or equivalent isotropic displacement parameters (Å^2^)

**Table:**

	*x*	*y*	*z*	*U*_iso_*/*U*_eq_	
Cr1	0.0000	0.148676 (17)	0.2500	0.02141 (14)	
O1	0.10432 (13)	0.15066 (6)	0.5670 (2)	0.0278 (3)	
H1A	0.1841 (11)	0.1603 (11)	0.618 (4)	0.038 (6)*	
H1B	0.097 (2)	0.1194 (8)	0.626 (3)	0.039 (6)*	
O2	0.11598 (12)	0.22143 (6)	0.2606 (2)	0.0273 (3)	
O3	0.10971 (12)	0.07688 (6)	0.2448 (2)	0.0275 (3)	
O4	0.12757 (13)	0.33040 (6)	0.2756 (2)	0.0324 (3)	
O5	0.11585 (14)	−0.03196 (7)	0.2324 (2)	0.0394 (4)	
C1	0.06468 (17)	0.01913 (8)	0.2430 (3)	0.0259 (4)	
C2	0.07080 (16)	0.27821 (8)	0.2608 (3)	0.0236 (3)	
N11	0.5000	0.15711 (11)	0.2500	0.0374 (5)	
N12	0.5000	−0.04417 (12)	0.2500	0.0393 (6)	
H12	0.5000	−0.0864	0.2500	0.096 (17)*	
C11	0.3808 (3)	0.19340 (12)	0.2074 (5)	0.0594 (7)	
H11C	0.3998	0.2396	0.2191	0.121 (15)*	
H11D	0.3548	0.1811	0.3077	0.088 (11)*	
H11E	0.3087	0.1836	0.0671	0.077 (10)*	
C12	0.5000	0.09124 (12)	0.2500	0.0263 (5)	
C13	0.38704 (18)	0.05453 (10)	0.2186 (3)	0.0341 (4)	
H13	0.3097	0.0760	0.1967	0.049 (7)*	
C14	0.3915 (2)	−0.01145 (10)	0.2204 (3)	0.0394 (5)	
H14	0.3167	−0.0349	0.2005	0.070 (9)*	

### Atomic displacement parameters (Å^2^)

**Table:**

	*U*^11^	*U*^22^	*U*^33^	*U*^12^	*U*^13^	*U*^23^
Cr1	0.0191 (2)	0.0153 (2)	0.0318 (2)	0.000	0.01434 (16)	0.000
O1	0.0272 (7)	0.0222 (6)	0.0332 (7)	−0.0025 (5)	0.0147 (6)	0.0010 (5)
O2	0.0237 (6)	0.0200 (6)	0.0417 (7)	−0.0003 (4)	0.0192 (6)	0.0007 (5)
O3	0.0236 (6)	0.0222 (6)	0.0400 (7)	0.0021 (5)	0.0186 (5)	−0.0004 (5)
O4	0.0275 (6)	0.0205 (6)	0.0477 (8)	−0.0031 (5)	0.0180 (6)	0.0019 (5)
O5	0.0379 (8)	0.0254 (7)	0.0459 (8)	0.0093 (5)	0.0148 (6)	−0.0056 (6)
C1	0.0242 (8)	0.0219 (8)	0.0252 (8)	0.0029 (6)	0.0080 (7)	−0.0011 (6)
C2	0.0216 (8)	0.0216 (8)	0.0258 (8)	0.0007 (6)	0.0110 (6)	0.0014 (6)
N11	0.0452 (14)	0.0244 (11)	0.0443 (14)	0.000	0.0240 (12)	0.000
N12	0.0497 (15)	0.0244 (12)	0.0397 (13)	0.000	0.0198 (11)	0.000
C11	0.0716 (18)	0.0393 (13)	0.0646 (16)	0.0220 (12)	0.0327 (15)	−0.0016 (11)
C12	0.0265 (12)	0.0271 (12)	0.0259 (11)	0.000	0.0138 (10)	0.000
C13	0.0248 (9)	0.0395 (10)	0.0398 (10)	−0.0006 (7)	0.0178 (8)	−0.0021 (8)
C14	0.0376 (11)	0.0398 (11)	0.0399 (11)	−0.0139 (8)	0.0190 (9)	−0.0035 (8)

### Geometric parameters (Å, °)

**Table:**

Cr1—O3^i^	1.9466 (13)	N11—C12	1.342 (3)
Cr1—O3	1.9466 (13)	N11—C11^ii^	1.447 (3)
Cr1—O2	1.9706 (13)	N11—C11	1.447 (3)
Cr1—O2^i^	1.9706 (13)	N12—C14^ii^	1.333 (3)
Cr1—O1^i^	2.0067 (14)	N12—C14	1.333 (3)
Cr1—O1	2.0067 (14)	N12—H12	0.8600
O1—H1A	0.821 (10)	C11—H11C	0.9600
O1—H1B	0.802 (10)	C11—H11D	0.9600
O2—C2	1.269 (2)	C11—H11E	0.9600
O3—C1	1.283 (2)	C12—C13	1.414 (2)
O4—C2	1.224 (2)	C12—C13^ii^	1.414 (2)
O5—C1	1.218 (2)	C13—C14	1.345 (3)
C1—C1^i^	1.546 (4)	C13—H13	0.9300
C2—C2^i^	1.557 (3)	C14—H14	0.9300
			
O3^i^—Cr1—O3	82.57 (8)	O4—C2—C2^i^	119.61 (10)
O3^i^—Cr1—O2	177.08 (5)	O2—C2—C2^i^	114.16 (9)
O3—Cr1—O2	97.57 (6)	C12—N11—C11^ii^	120.73 (15)
O3^i^—Cr1—O2^i^	97.57 (6)	C12—N11—C11	120.73 (15)
O3—Cr1—O2^i^	177.08 (5)	C11^ii^—N11—C11	118.5 (3)
O2—Cr1—O2^i^	82.45 (7)	C14^ii^—N12—C14	120.0 (3)
O3^i^—Cr1—O1^i^	91.57 (5)	C14^ii^—N12—H12	120.0
O3—Cr1—O1^i^	90.16 (5)	C14—N12—H12	120.0
O2—Cr1—O1^i^	91.35 (5)	N11—C11—H11C	109.5
O2^i^—Cr1—O1^i^	86.91 (5)	N11—C11—H11D	109.5
O3^i^—Cr1—O1	90.16 (5)	H11C—C11—H11D	109.5
O3—Cr1—O1	91.57 (5)	N11—C11—H11E	109.5
O2—Cr1—O1	86.91 (5)	H11C—C11—H11E	109.5
O2^i^—Cr1—O1	91.35 (5)	H11D—C11—H11E	109.5
O1^i^—Cr1—O1	177.70 (7)	N11—C12—C13	121.92 (12)
Cr1—O1—H1A	114.1 (17)	N11—C12—C13^ii^	121.92 (12)
Cr1—O1—H1B	116.7 (17)	C13—C12—C13^ii^	116.2 (2)
H1A—O1—H1B	110 (2)	C14—C13—C12	119.97 (19)
C2—O2—Cr1	114.53 (11)	C14—C13—H13	120.0
C1—O3—Cr1	115.20 (11)	C12—C13—H13	120.0
O5—C1—O3	125.28 (17)	N12—C14—C13	121.95 (19)
O5—C1—C1^i^	121.26 (11)	N12—C14—H14	119.0
O3—C1—C1^i^	113.46 (9)	C13—C14—H14	119.0
O4—C2—O2	126.23 (15)		
			
O3^i^—Cr1—O2—C2	−89.0 (10)	Cr1—O3—C1—C1^i^	−3.0 (2)
O3—Cr1—O2—C2	178.46 (12)	Cr1—O2—C2—O4	176.72 (14)
O2^i^—Cr1—O2—C2	1.40 (9)	Cr1—O2—C2—C2^i^	−3.4 (2)
O1^i^—Cr1—O2—C2	88.11 (12)	C11^ii^—N11—C12—C13	177.00 (16)
O1—Cr1—O2—C2	−90.37 (12)	C11—N11—C12—C13	−3.00 (16)
O3^i^—Cr1—O3—C1	1.22 (9)	C11^ii^—N11—C12—C13^ii^	−3.00 (16)
O2—Cr1—O3—C1	178.27 (12)	C11—N11—C12—C13^ii^	177.00 (16)
O2^i^—Cr1—O3—C1	−91.7 (10)	N11—C12—C13—C14	−179.79 (14)
O1^i^—Cr1—O3—C1	−90.34 (12)	C13^ii^—C12—C13—C14	0.21 (14)
O1—Cr1—O3—C1	91.18 (12)	C14^ii^—N12—C14—C13	0.22 (15)
Cr1—O3—C1—O5	177.34 (15)	C12—C13—C14—N12	−0.4 (3)

Symmetry codes: (i) −*x*, *y*, −*z*+1/2; (ii) −*x*+1, *y*, −*z*+1/2.

### Hydrogen-bond geometry (Å, °)

**Table:**

*D*—H···*A*	*D*—H	H···*A*	*D*···*A*	*D*—H···*A*
O1—H1A···O4^iii^	0.82 (1)	1.91 (1)	2.719 (2)	171 (2)
O1—H1B···O5^iv^	0.80 (1)	1.91 (1)	2.680 (2)	160 (2)
N12—H12···O4^v^	0.86	2.19	2.906 (3)	141
N12—H12···O4^vi^	0.86	2.19	2.906 (3)	141

Symmetry codes: (iii) −*x*+1/2, −*y*+1/2, −*z*+1; (iv) *x*, −*y*, *z*+1/2; (v) −*x*+1/2, *y*−1/2, −*z*+1/2; (vi) *x*+1/2, *y*−1/2, *z*.
